# Supplementary material for: Modeling glioblastoma heterogeneity as a dynamic network of cell states
Source: Mol Syst Biol. 2021 Sep 16;17(9):e10105. doi: 10.15252/msb.202010105 (PMC8444284; doi:10.15252/msb.202010105)
Supplement: Supplementary file 6 — Source Data for Figure 5 [file MSB-17-e10105-s004.zip › Figure5A_sourcedata/GSEA_3017/nosigdb_stateC.GseaPreranked.1622795002785/NB_KI_CLUSTER17.html]

Details for gene set NB\_KI\_CLUSTER17[GSEA]

|  || Dataset | state13017 |
| Phenotype | NoPhenotypeAvailable |
| Upregulated in class | na\_neg |
| GeneSet | NB\_KI\_CLUSTER17 |
| Enrichment Score (ES) | -0.5212039 |
| Normalized Enrichment Score (NES) | -2.644742 |
| Nominal p-value | 0.0 |
| FDR q-value | 0.0 |
| FWER p-Value | 0.0 |
Table: GSEA Results Summary

  

Fig 1: Enrichment plot: NB\_KI\_CLUSTER17      
 Profile of the Running ES Score & Positions of GeneSet Members on the Rank Ordered List

  

| PROBE | GENE SYMBOL | GENE\_TITLE | RANK IN GENE LIST | RANK METRIC SCORE | RUNNING ES | CORE ENRICHMENT || 1 | UGCG |  |  | 287 | -0.273 | -0.4859 | No |
| 2 | PLEKHA4 |  |  | 308 | -0.281 | -0.4994 | Yes |
| 3 | PDLIM2 |  |  | 314 | -0.283 | -0.4862 | Yes |
| 4 | AHR |  |  | 324 | -0.286 | -0.4799 | Yes |
| 5 | SPATS2L |  |  | 339 | -0.290 | -0.4821 | Yes |
| 6 | FTH1 |  |  | 357 | -0.301 | -0.4888 | Yes |
| 7 | LRP1 |  |  | 366 | -0.304 | -0.4793 | Yes |
| 8 | SPRY1 |  |  | 369 | -0.307 | -0.4589 | Yes |
| 9 | PMEPA1 |  |  | 375 | -0.308 | -0.4438 | Yes |
| 10 | CREB3L2 |  |  | 378 | -0.309 | -0.4234 | Yes |
| 11 | RHOB |  |  | 391 | -0.316 | -0.4200 | Yes |
| 12 | S100A13 |  |  | 396 | -0.319 | -0.4023 | Yes |
| 13 | REXO2 |  |  | 432 | -0.342 | -0.4376 | Yes |
| 14 | WWTR1 |  |  | 437 | -0.345 | -0.4178 | Yes |
| 15 | RHOC |  |  | 455 | -0.359 | -0.4199 | Yes |
| 16 | FAM126A |  |  | 456 | -0.360 | -0.3919 | Yes |
| 17 | EDNRB |  |  | 462 | -0.367 | -0.3723 | Yes |
| 18 | HES1 |  |  | 470 | -0.373 | -0.3556 | Yes |
| 19 | RAB13 |  |  | 482 | -0.388 | -0.3449 | Yes |
| 20 | JUN |  |  | 503 | -0.425 | -0.3472 | Yes |
| 21 | AHNAK |  |  | 519 | -0.449 | -0.3388 | Yes |
| 22 | PLPP3 |  |  | 528 | -0.466 | -0.3168 | Yes |
| 23 | VMP1 |  |  | 542 | -0.502 | -0.3007 | Yes |
| 24 | RCAN1 |  |  | 544 | -0.505 | -0.2633 | Yes |
| 25 | TNFRSF12A |  |  | 550 | -0.519 | -0.2318 | Yes |
| 26 | PLAT |  |  | 556 | -0.538 | -0.1988 | Yes |
| 27 | IL1RAP |  |  | 558 | -0.544 | -0.1583 | Yes |
| 28 | ANXA2 |  |  | 563 | -0.567 | -0.1212 | Yes |
| 29 | LAMA4 |  |  | 571 | -0.620 | -0.0854 | Yes |
| 30 | EMP1 |  |  | 589 | -0.785 | -0.0544 | Yes |
| 31 | PMP22 |  |  | 590 | -0.837 | 0.0106 | Yes |
Table: GSEA details [plain text format]

  

Fig 2: NB\_KI\_CLUSTER17: Random ES distribution      
 Gene set null distribution of ES for **NB\_KI\_CLUSTER17**
